# High Throughput miRNA Screening Identifies miR-574-3p Hyperproductive Effect in CHO Cells

**DOI:** 10.3390/biom11081125

**Published:** 2021-07-30

**Authors:** Živa Švab, Luca Braga, Corrado Guarnaccia, Ivan Labik, Jeremias Herzog, Marco Baralle, Mauro Giacca, Nataša Skoko

**Affiliations:** 1 Biotechnology Development Unit, International Centre for Genetic Engineering and Biotechnology (ICGEB), 34149 Trieste, Italy; Ziva.Marsetic@icgeb.org (Ž.Š.); guarna@icgeb.org (C.G.); 2 Functional Cell Biology Group, International Centre for Genetic Engineering and Biotechnology (ICGEB), 34149 Trieste, Italy; braga@icgeb.org; 3 RNA Biology Group, International Centre for Genetic Engineering and Biotechnology (ICGEB), 34149 Trieste, Italy; ivan.labik@outlook.com (I.L.); herzogjeremias@gmail.com (J.H.); barallem@icgeb.org (M.B.); 4 Molecular Medicine Group, International Centre for Genetic Engineering and Biotechnology (ICGEB), 34149 Trieste, Italy; giacca@icgeb.org; 5 British Heart Foundation Centre of Research Excellence, School of Cardiovascular Medicine & Sciences, King’s College London, London SE5 9NU, UK

**Keywords:** miRNA screening, miR-574-3p, hyperproductivity, CHO, pri-miRNA processing, p300, p53

## Abstract

CHO is the cell line of choice for the manufacturing of many complex biotherapeutics. The constant upgrading of cell productivity is needed to meet the growing demand for these life-saving drugs. Manipulation of small non-coding RNAs—miRNAs—is a good alternative to a single gene knockdown approach due to their post-transcriptional regulation of entire cellular pathways without posing translational burden to the production cell. In this study, we performed a high-throughput screening of 2042-human miRNAs and identified several candidates able to increase cell-specific and overall production of Erythropoietin and Etanercept in CHO cells. Some of these human miRNAs have not been found in Chinese hamster cells and yet were still effective in them. We identified miR-574-3p as being able, when overexpressed in CHO cells, to improve overall productivity of Erythropoietin and Etanercept titers from 1.3 to up to 2-fold. In addition, we validated several targets of miR-574-3p and identified p300 as a main target of miR-574-3p in CHO cells. Furthermore, we demonstrated that stable CHO cell overexpressing miRNAs from endogenous CHO pri-miRNA sequences outperform the cells with human pri-miRNA sequences. Our findings highlight the importance of flanking genomic sequences, and their secondary structure features, on pri-miRNA processing offering a novel, cost-effective and fast strategy as a valuable tool for efficient miRNAs engineering in CHO cells.

## 1. Introduction

Chinese Hamster Ovary (CHO) cells are the most frequently used mammalian cells in biopharmaceutical manufacturing [1]. CHO cells can reach high volumetric and cell specific productivity and can easily be adapted to grow in suspension in chemically defined media which is the method of choice for large-scale industrial production [2,3]. Furthermore, CHO cells are devoid of human viruses, giving them an additional advantage for commercial production purposes in terms of biosafety risks reduction. Therefore, many biologics produced in CHO cells have been approved for human use, which makes them additionally appealing from the registration point of view. However, industry is constantly seeking means to obtain improved and more profitable CHO cell line derivatives in order to meet growing demands for biologics and to lower the costs of production, which can lead to more affordable therapeutics [4,5]. 

Among new CHO cell engineering technologies, small noncoding molecules such as miRNAs represent an attractive target as gene expression regulators. These are 17–23 nucleotide long, single-stranded, evolutionarily conserved RNA molecules that mediate post-transcriptional gene regulation through a mechanism that involves RISC-mediated binding to complementary sequences in the 3’ UTR of target mRNAs. As only the short seed sequence (2–8 nt from the 5’ end) is responsible for target binding, a single miRNA can target different sequences, regulating the expression of multiple genes [6]. miRNAs regulate almost all cellular processes in metazoans as a switch and a fine-tuner of gene expression through target mRNAs translational repression, destabilization or degradation [7,8,9,10,11]. Major advantages in miRNA engineering of industrially important CHO cell line are their ability to target multiple mRNAs and to place no additional burden on the translational machinery [12]. The whole CHO genome sequence paves the way for research on miRNAs’ expression profiles and the identification of miRNAs gene location in different CHO cell lines [12,13,14,15,16,17,18,19]. Most of the miRNAs detected in CHO cells are conserved and have validated targets in mice, humans, and rats [19].

The first report of the application of miRNAs in CHO cell line engineering was published by Barron et al. where transient overexpression of miR-7 enhanced cell-specific productivity with a negative effect on viable cell content [20]. So far, just a few miRNAs, when overexpressed, were able to enhance recombinant protein production in CHO cells. These include miR-466h [21,22], miR-17, -19b and 92a [23], miR-557 and miR-1287 [24], miR-17 [25], miR-483 [26], miR-30 family [27], miR-2861 [28], miR-143 [29], miR-31 [30] and miR-200a [31]. On the other hand, downregulation or knock-out of miRNAs, such as miR-23b [32] or miR-744-3p [33], increased recombinant protein expression as well.

The aim of this study was to screen a human genome miRNA library for their ability to increase recombinant protein production in CHO cells in a product-independent manner. Using a human library opens up the possibility of identifying miRNAs not previously found in Chinese hamsters that may have conserved targets and functions in CHO cells. In fact, this study identified several new process relevant miRNAs for the production of two recombinant proteins of pharmaceutical interest, Erythropoietin (EPO) and Etanercept (ETN). In particular, we showed that miR-574-3p is able to enhance CHO cell productivity by increasing transcription and inhibiting apoptosis. By molecular characterization of this process relevant miRNA action, we validated several target mRNAs and identified p300 as the main miR-574-3p effector protein. Finally, we showed that p53 might be a potential downstream target gene of miR-574-3p in CHO cells, which might be one of the mechanisms of its pro-productive activity.

We also compared the effect of Chinese hamster and human flanking sequences on miRNAs biogenesis in CHO cells. This work offers a novel strategy for the stable expression of any miRNA in CHO cells through site-directed mutagenesis of hamster-specific flanking sequences of precursor miRNA (pri-miR)-143. It also highlights the importance of miRNAs secondary structure features for mature miRNA biogenesis in CHO cells. 

## 2. Material and Methods

### 2.1. CHO-EPO and CHO-ETN Stable Clones Preparation

Adherent CHO cells expressing human EPO and ETN were made using CHO-DUKX-B11 (dhfr-) cells. The human erythropoietin gene was obtained from genomic human DNA by PCR and cloned into a eukaryotic expression vector pBT-5 under the control of SV40 promotor. Etanercept was cloned into the pOptiVEC vector under the CMV promoter. Methotrexate at 0.1 µm was used in order to select the best producing single clones. CHO-EPO and CHO-ETN cell lines were grown in GlutaMAX™ DMEM high glucose media (Gibco, Grand Island, NY, USA) supplemented with 10% FBS. For the production phase, the serum was depleted from the media and DMEM media was supplemented with 5 µg/mL insulin and 50 mg/mL dextrane sulphate 5000. 

### 2.2. miRNA Mimics Screening

A library of 2042 miRIDIAN human microRNA mimics (miRBASE, version 19.0, GE Healthcare Dharmacon, Lafayette, CO, USA) was reverse-transfected into CHO-EPO cells (50 nM final concentration) using automated liquid handling workstation, Microlab STAR Line (Hamilton, Bondauz, Switzerland). CHO-EPO cells were seeded as 1500 cell/well in 384-well format in DMEM supplemented with 10% FBS in wells already containing miRNA mimics and lipofectamine (RNAiMAX, ThermoFisher, Waltham, MA, USA). After 24 h at 37 °C, media was replaced with DMEM without FBS. After 24 h, media was recovered and the amount of EPO quantified by ELISA assay. Viable cell density was determined after fixing of the cells and staining with Hoechst 33342 stain (Invitrogen, ThermoFischer Scientific, Waltham, MA, USA). Data analysis was done using MetaXpress v.5.1 (Molecular Devices, Sunnyvale, CA, USA). Scaled-up transient transfection experiments were done in 96-well culture treated plates with 7500 cell/well. 

### 2.3. Stable miRNAs Expression Clones Preparation

Native pri-miR sequences of *Homo sapiens* (hsa) and *Cricetulus griseus* (cgr) and its flanking regions were obtained by PCR from human or CHO genomic DNA, respectively, using primers located cca. 200 nt upstream and downstream from the miRNA genomic location. The pri-miR sequence information was extracted from miRBase (Release 21) and the Ensemble genome browser (www.ensembl.org, accessed on 4 December 2017, release 89). PCR was performed using *Pfu* polymerase (Promega, Madison, WI, USA). To obtain suitable negative control (CHO-EPO neg. ctrl. and CHO-ETN neg. ctrl.), an insert from the pcDNA™6.2-GW/± EmGFP-miR-neg control plasmid (Invitrogen) that can form a hairpin structure and is processed into mature miRNA, but is predicted not to target any known vertebrate gene, has been PCR amplified. All primers are listed in Supplementary Table S1. PCR fragments were ligated into the miRVec expression vector (pMSCV-blasticidin vector, Voorhoeve et al., 2007). With the site-directed mutagenesis approach, the cassette coding for mature miRNA-143-3p inside cgr-pri-miR-143 sequence was exchanged with the selected mature miRNAs within miRVec plasmid using primers listed in the Supplementary Table S2. Primers for the hsa-pri/pre-miR, designed to amplify the entire stem-loop of pre-miRNA, are listed in the Supplementary Table S4. Total RNA was extracted from cells using miRNeasy mini Kit (Qiagen, Düsseldorf, Germany) and reverse transcribed using miRCURY LNA RT Kit (Qiagen) which was then used for qPCR using miRCURY LNA SYBR Green PCR Kit (Qiagen). Primers to detect miRNA levels were miRNA LNA PCR primer sets (miRCURY LNA miRNA PCR Assays, Qiagen). MiRNA expression is depicted as fold-change relative to the parental CHO control cells and normalized to miR-191-5p. In accordance with Peltier HJ and Latham GJ, we demonstrated that miR-191 has a highly consistent expression [34].

### 2.4. Real-Time PCR

For the determination of mRNA levels of miR-574-3p targets, on-column DNA digestion was performed using RNase-Free DNase Set (Qiagen) during RNA extraction. The RNA was retrotranscribed with Moloney murine leukemia virus (M-MLV) reverse transcriptase and Oligo(dT)12–18 primers. cDNA was used as template for Rt-PCR using SYBRGreen technology (Biorad, Hercules, CA, USA). Housekeeping genes MMADHC and GAPDH were used for normalization. A complete list of primers used for qRT-PCR analysis are provided in the Supplementary Table S3. All amplifications were done on CFX96 real-time PCR detection system (Biorad). Relative mature miRNA expression differences were calculated by applying the comparative C(T) method [35].

### 2.5. EPO Purification 

Medium with EPO collected during 12 days of cultivation was filtered and purified using affinity resin Blue Sepharose in HiTrap Blue HP (GE Healthcare Life Sciences, Marlborough, MA, USA) column and anion exchange high performance resin HiTrap-Q in HiTrap Q HP 5 mL (GE) column interposed by two desalting steps using a column prepacked with Sephadex G25 Fine.

### 2.6. Western Blot Analysis

For Western Blot analysis, cells were lysed in Lysis buffer (1X PBS, 1 mM Na_2_VO_4_ and 2 X complete Mini EDTA-free Protease Inhibitor Cocktail (Roche Diagnostic, Basel, Switzerland) by 2 min sonication. Total protein concentration was determined by Bradford and 20–30 μg/well of total proteins were separated on SDS-PAGE TruPAGE Precast Gels 4–12% (Sigma, St. Louis, MI, USA) and transferred onto a nitrocellulose membrane. The following antibodies were used for Western: Anti-p300 (mouse, 1:200, Santa Cruz Biotechnology #sc-32244), Anti-TGFβ1 (mouse, 1:200, Santa Cruz Biotechnology #sc-130348), Anti-SMAD4 (mouse, 1:200, Santa Cruz Biotechnology #sc-7966), Anti-Bcl-Xl (mouse, 1:200, Santa Cruz Biotechnology #sc-8392), Anti-RXRα (mouse, 1:100, Santa Cruz Biotechnology #sc-515929), Anti-clathrin HC (mouse, 1:200, Santa Cruz Biotechnology #sc-12734), Anti-ERH (mouse, 1:100, Santa Cruz Biotechnology #sc-373906), Anti-p53 DO1 (mouse, 1:200 Santa Cruz Biotechnology #sc-126), Anti-p53 Pab240 (mouse, 1:500, Santa Cruz Biotechnology #sc-99), Anti-Tubulin (mouse, 1:1000, Sigma-Aldrich ##T6199), Anti-GAPDH (mouse, 1:7000, Sigma-Aldrich #G9295) and the secondary antibody HRP-labeled anti-mouse antibody (1:1000, Dako #P0447) and HRP-labeled anti-rabbit antibody (1:1000, Dako #P0448). Protein detection was assessed on the Alliance chemiluminescence imaging system (Uvitec) using ECL Western Blotting Substrate (ThermoFischer Scientific, Waltham, MA, USA). Protein bands were quantified using NIH ImageJ. 

### 2.7. Iso-Electric Focusing

Iso-electrofocusing gels were prepared as detailed (3.15 g of Urea, 2.1 mL of 30% acrylamide/bisacrylamide (Protogel, National Diagnostics, Nottingham, UK), 0.35 mL of pH = 2.5 to 5 ampholyte (GE), 0.175 mL of pH = 3 to 10 ampholyte (GE), 4.725 mL of ultrapure water, 5.25 µL of TEMED, 105 µL of 100 g/L APS. As an anolyte buffer 0.1 M glutamic acid, 0.5 M phosphoric acid was used and as a catholyte buffer 0.89% beta-alanine. The gel was pre-run for 60 min at a constant power of 5 W, with maximum amperage of 50 mA. The focusing was carried out for further 180 min at a constant power of 7 W, with maximum amperage of 50 mA. After separation, the gel was placed in the in equilibration buffer (125 mM Tris pH = 6.8, 5% 2-mercaptoethanol, 1% SDS) for 20 min at 4 °C and subsequently into transfer buffer (25 mM Tris, 192 mM Glycine) for 20 min at 4 °C. Separated EPO isoforms were then transferred to PVDF membrane and probed with primary anti-EPO antibody a 1:1000 dilution (rabbit anti-EPO R&D) for 1 h at room temperature followed by incubation with anti-rabbit (HRP-conjugate) secondary antibody diluted at 1:2000 (ThermoFischer Scientific) for additional 1 h at room temperature. 

### 2.8. siRNA Transfections

CHO cells were grown in 12 well plate dishes in standard Dulbecco’s Modified Eagle Medium (DMEM) supplemented with 10% FBS. Cells were approximately 50–60% confluent before being transfected with 2 nM siRNA using Lipofectamine RNAiMAX (Invitrogen) according to manufacturer’s instructions. Cells were harvested 72 h after transfection. SiRNA transfection efficiency was determined by real-time RT-PCR and Western Blot analysis. Oligonucleotides for siRNA generation are listed in Supplementary Table S5. 

## 3. Results

### 3.1. High-Throughput miRNA Mimics Screening in CHO-EPO and CHO-ETN Cells

**Figure 1:**
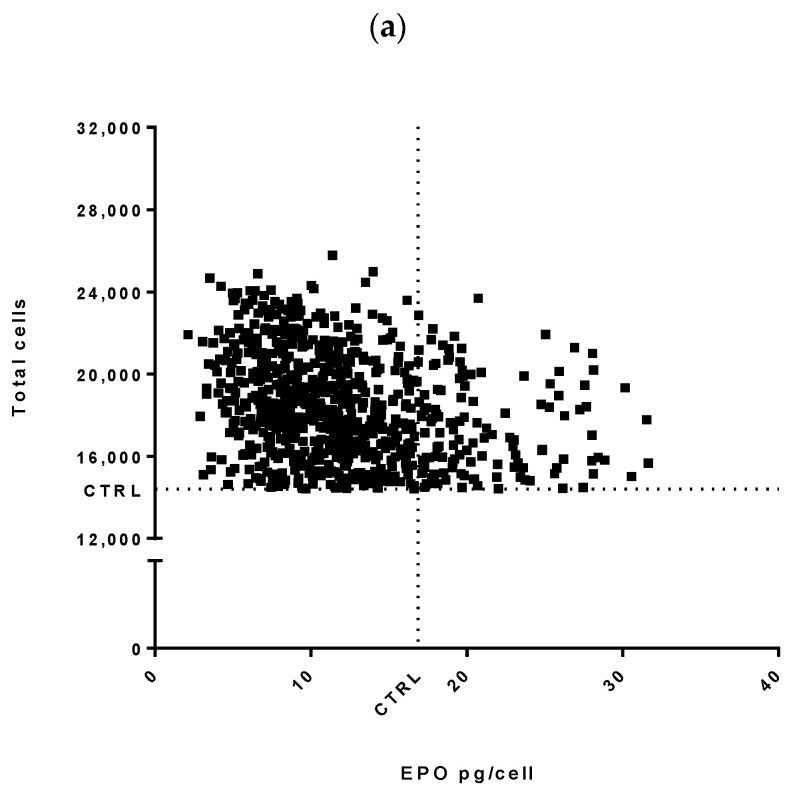

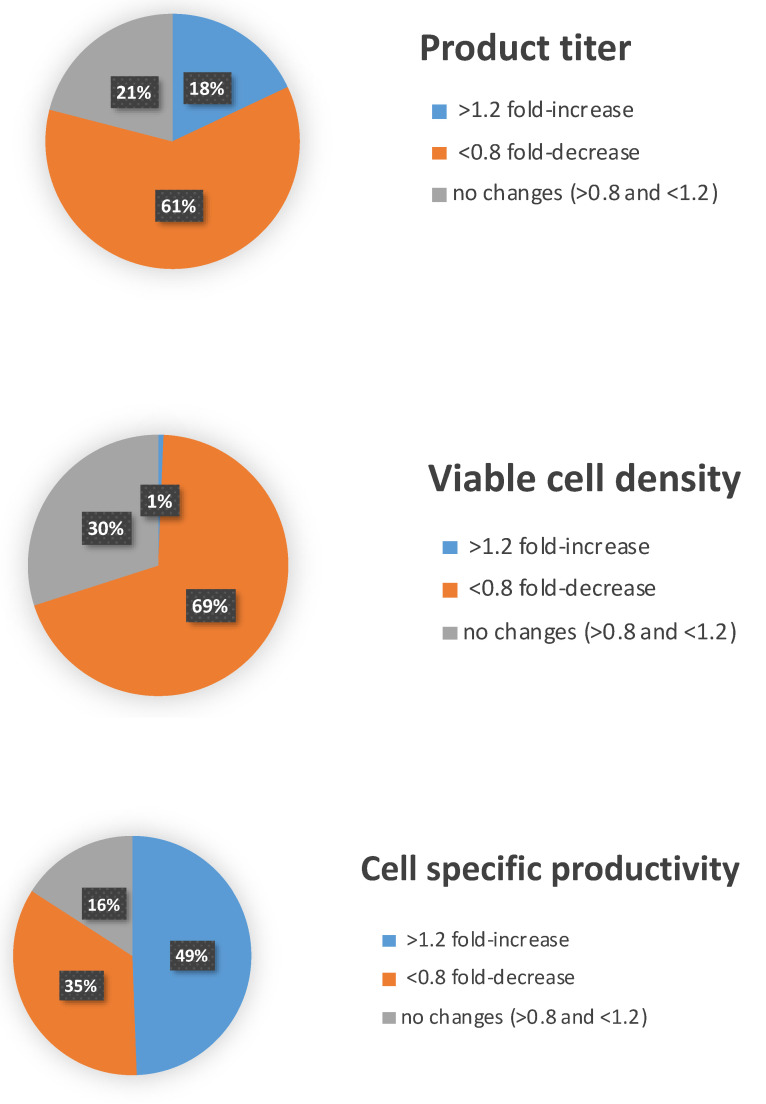

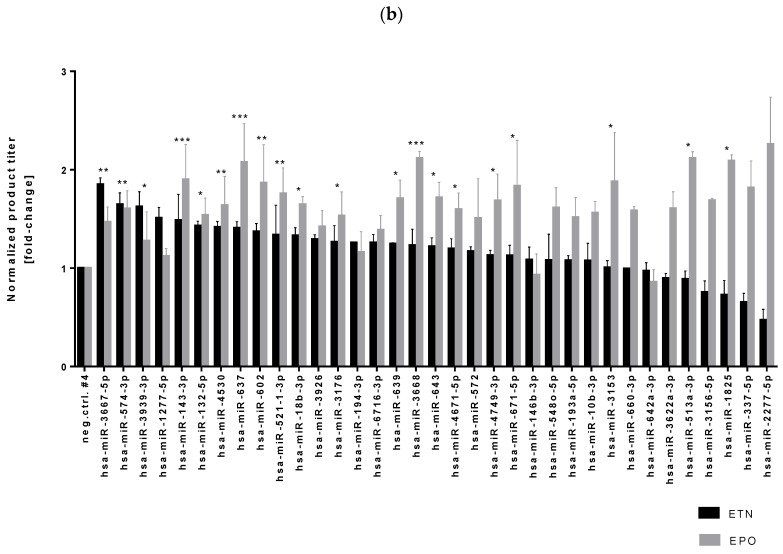
A functional high-throughput miRNA mimic screening in CHO-EPO and CHO-ETN cells. (**a**) Overview of the normalized results of 2042 miRNA mimics on the recombinant CHO-EPO cells. EPO titer, cell specific EPO productivity and viable cell density are represented as fold-changes with respect to the negative control. The pie charts illustrate the percentages of miRNA mimics that induce at least a 1.2-fold increase to at least a 0.8-fold decrease. (**b**) The effect of selected 35 miRNA mimics transiently transfected in CHO-EPO and CHO-ETN cells. Normalized volumetric productivities are presented as fold-changes relative to the respective negative control. Data is presented as the mean of 3 independent experiments ± SEM. Statistical analysis was performed using two-way ANOVA with Fisher’s analysis (* *p* ≤ 0.05, ** *p* ≤ 0.01 and *** *p* ≤ 0.001).

A screen of 2042 human miRNA mimics (miRIDIAN microRNA mimics Dharmacon, miRbase release V19.0), transiently transfected into the CHO-EPO cell line, aimed to identify miRNAs able to boost recombinant protein production. These RNA molecules were designed to mimic endogenous mature miRNA molecules and were introduced into cells via reverse transfection using an automated liquid handling workstation (Microlab STAR Line, Hamilton, Reno, NV, USA). We used a series of controls including mock-transfected cells, two non-targeting control miRNA mimics and toxic siRNA targeting the Ubiquitin C (UBC) transcript to optimize the screen in a 384-well plates format. Seventy-two hours post-transfection, we measured a range of bioprocess relevant parameters such as viable cell density, product titer and specific cell productivity. Viable cell density was increased by 14 miRNAs while 615 miRNAs had no effect on cell proliferation. 1015 miRNAs enhanced cell-specific EPO productivity, whilst 370 miRNAs resulted in higher volumetric productivity (Figure 1a).

In order to validate the high-throughput screening results and identify miRNAs that increase recombinant protein production in CHO cells in a product-independent manner, we chose 124 miRNAs with the highest product titer and viable cell density for a secondary screening in CHO-EPO and CHO-ETN cells (Supplementary Figure S1). Next, we scaled-up our experiment to a 96-well plate format with both CHO-EPO and CHO-ETN cells transiently transfected with 35 best-performing miRNA mimics. We selected seven miRNA mimics (miR-143-3p, miR-18b-3p, miR-132-5p, miR-521-1-3p, miR-574-3p, miR-3667-5p and miR-3939-3p), which significantly improved both EPO and ETN production in CHO cells, for further studies (Figure 1b).

### 3.2. Stable Expression of Selected miRNAs in CHO Cells

Successful use of miRNAs as tools for genetic optimization of CHO producer cells in industrial applications relies on the stable expression of miRNAs through the integration of the pre-miRNA sequence with upstream and downstream flanking regions into the host genome. We were able to amplify six out of seven top performing pri-miRNAs from human genomic DNA using primers located around 200 nt upstream and 200 nt downstream of pre-miRNAs. These were cloned under the CMV promoter in the miRVec vector. After stable clone selection, we confirmed the presence of human pri-miRNA-143, pri-miRNAs-18b, pri-miRNA-521, pri-miRNA-574, pri-miRNA-3667 and pri-miRNA-3939 in the CHO genome by PCR analysis (Supplementary Figure S2a). 

**Figure 2:**
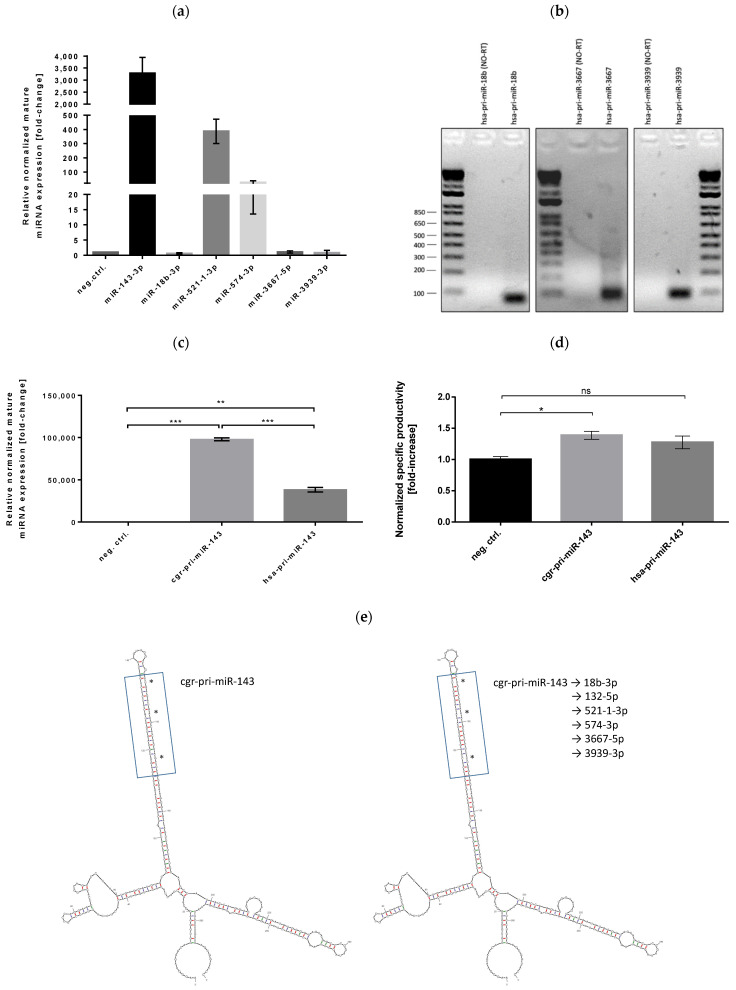
(**a**) Analysis of the relative level of mature miRNAs in stable CHO-ETN cells expressed from human pri-miRNAs. MiRNAs expression is shown as fold-change relative to the CHO-ETN control cells transduced with non-targeting miRNA and normalized to miR-191-5p. Data is presented as the mean of three independent experiments ± SEM (**b**) RT-PCR analyses of the total RNA extracted from CHO-ETN stable clones overexpressing hsa-miR-18b/3667/3939 confirmed the presence of the precursor miRNA transcripts in the CHO cells (**c**) Analysis of the relative level of mature miRNAs in stable CHO-ETN cells expressed from different pri-miRNA scaffolds. MiRNAs expression is shown as fold-change relative to the CHO-ETN control cells transduced with non-targeting miRNA and normalized to miR-191-5p (**d**) Effects of stable overexpression of miR-143 on ETN production. Data is presented as the mean of 3 independent experiments ± SEM. Statistical analysis was done using one-way ANOVA followed by Bonferroni’s post-hoc test (ns: not significant, * *p* ≤ 0.05, ** *p* ≤ 0.01 and *** *p* ≤ 0.001). (**e**) Predicted secondary structure of pri-miRNAs of interest shows a hairpin, double-stranded stem and three bulges located within the miRNA stem. The most probable secondary structure of pri-miRNAs transcripts was obtained using UnaFold software.

Analysis of relative levels of mature miRNAs in stable CHO-ETN cells showed that only three out of six human pri-miRNAs were successfully produced in CHO cells (Figure 2a). Expression of mature miR-18b-3p, miR-3667-5p and miR-3939-3p was non detectable in CHO-ETN stable clones. In order to further investigate whether the problem was in the transcription or the processing of human pri-miRNAs, we performed RT-PCR analyses of the total RNA extracted from CHO-ETN–hsa-miR-18b/3667/3939 stable clones and confirmed the presence of the primary and precursor transcripts (Figure 2b). This result demonstrated that the transcription of human pri-miR-18b/3667/3939 was not impaired and that the problem lay in the processing of pri-miRNAs in CHO cells. One of the reasons for defective human pri-miRNAs processing in CHO cells could be the perturbation of the secondary structure, such as lack of formation of specific pri-miRNA hairpin. 

Knowing the complexity and importance of miRNAs flanking regions for mature miRNA biogenesis, the best way to generate the miRNA overexpressing stable CHO cell line would be to use an endogenous CHO scaffold. According to the miRBase database of published miRNA sequences, information on the chromosomal location of the miRNA genes in CHO cells was possible for only three out of the seven selected miRNAs. Therefore, we decided to compare human and Chinese hamster pri-miRNA-143 scaffolds in order to find the best flanking region to generate miRNA overexpressing stable CHO cells for all the seven selected miRNAs. The conservation rate of mature miR-143 in Chinese hamster sequence compared to human is 100% whereas precursor (pri-miR-143) sequences show significant differences (Supplementary Figure S2b). Chinese hamster endogenous pri-miR-143 (cgr-pri-miR-143) and human (hsa-pri-miR-143) were amplified, cloned into miRVec plasmid and transduced into CHO-ETN cells. 

The expression of mature miR-143 was confirmed by RT-PCR for all stable miRNA expressing CHO clones (Figure 2c). Being optimized for the Chinese hamster miRNA processing machinery, Chinese hamster endogenous pri-miRNA sequences result in higher mature miRNAs expression in comparison to miRNA precursor sequences from human genomic DNA. Significant increase in ETN production (1.4-fold) was only observed in the stable overexpression of miR-143 in CHO cells with an endogenous CHO scaffold (Figure 2d). These results demonstrate that the Chinese hamster pri-miRNA sequence is the method of choice for stable miRNA overexpression in CHO cells.

To date, several structural features have been identified to regulate pri-miRNA processing, including hairpin stem length of 36 ± 3 nt, apical loop size ≥10 nt and the location of unpaired bases along the stem (bulge position) [36,37,38]. These features are enriched in pri-miRNAs, discriminate them from other hairpin-containing transcripts and govern efficient processing. The double-stranded stem, hairpin structure and single-stranded flanking regions of pri-miRNA are critical for microprocessor complex binding and cleavage [39,40,41]. Several sequences, such as the CNNC and basal UG, have been shown to be processing-enhancing motifs in pri-miRNAs [36] but the overall view is that nucleotide identity has less impact on pri-miRNA processing than the secondary structure features [41,42].Taking all this into account, we decided to use Chinese hamster pri-miR-143 flanking sequences and replace the mature miR-143 encoding region with miR-18b, miR-132, miR-521, miR-574, miR-3667 and miR-3939 sequences in the miRVec plasmid. We applied site-directed mutagenesis maintaining hamster-specific flanking sequences of pri-miR-143, the length of the miR-143 stem, the location and size of three bulges as well as the sequence of the miRNA 3p strand while three sequence mismatches were introduced in the 5p strand. By mutating the opposite strands, we aimed to prevent their loading into Ago complexes and to promote “correct” strand incorporation by RISC. In this way, the most probable secondary structure of pri-miRNAs of interest, obtained using UnaFold software, shows a preserved hairpin, double-stranded stem and three bulges location within the miRNA stem (Figure 2e).

### 3.3. Successful Stable Expression of Seven miRNAs from Chinese Hamster pri-miR-143 Flanking Motifs in CHO Cells

**Figure 3:**
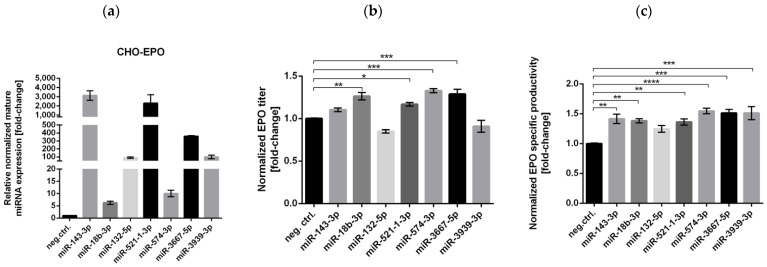

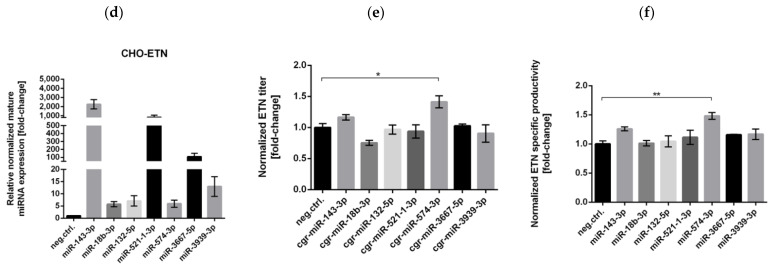
Effects of stable overexpression of seven miRNAs on EPO and ETN production. Relative mature miRNAs levels in (**a**) stable CHO-EPO expressing seven miRNAs and (**d**) stable CHO-ETN also expressing seven miRNAs shown as fold-change relative to the respective control cells and normalized to miR-191-5p. Normalized volumetric productivities of (**b**) EPO and (**e**) ETN are presented as a fold change to the respective negative control. Normalized specific productivities of (**c**) EPO and (**f**) ETN are presented as a fold change to the respective negative control. Data are presented as the means of three independent experiments ± SEM. Statistical analysis was performed using one-way ANOVA followed by Bonferroni’s post-hoc comparisons tests (* *p* ≤ 0.05, ** *p* ≤ 0.01, *** *p* ≤ 0.001 and **** *p* ≤ 0.0001).

The stable expression of all seven mature miRNAs of interest from Chinese hamster pri-miR-143 flanking motifs in CHO-EPO or ETN clones was confirmed by RT-PCR (Figure 3a,d). EPO and ETN concentration was measured in the supernatant after 4 days of production. While most of the tested miRNAs significantly increased the production of EPO, only miRNA-574-3p was able to increase CHO-ETN productivity. Stable overexpression of miRNA-574-3p resulted in a 1.33-fold increase of EPO titer and a 1.55-fold increase of cell-specific EPO productivity in CHO cells with endogenous CHO pri-miRNA scaffold (Figure 3b,c). Stable overexpression of miRNA-574-3p resulted in a 1.41-fold increase of ETN titer and a 1.48-fold increase of cell specific ETN productivity in CHO cells with endogenous CHO pri-miRNA scaffold (Figure 3e,f). This data provides valuable evidence for miR-574 as a cell-engineering target to enhance CHO cell productivity. 

**Figure 4:**
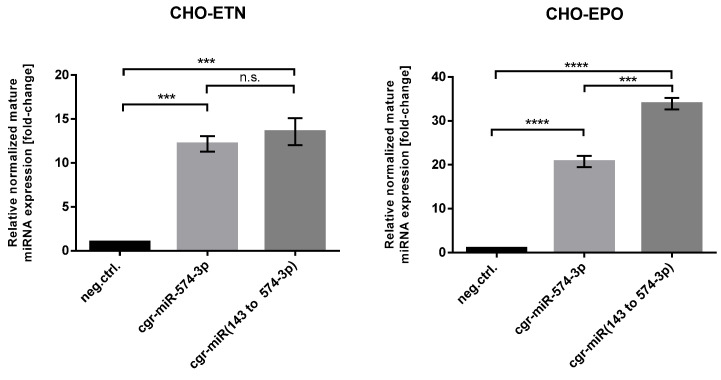
Comparison of relative mature miRNA-574-3p expression in CHO-ETN and CHO-EPO stable clones from pri-miR-574 and pri-miR-143 Chinese hamster flanking sequences. Quantitative RT-PCR analysis of the relative level of mature miRNAs shown as a fold-change relative to the respective control cells and normalized to miR-191-5p. Data are presented as mean ± SEM. Statistical analysis was performed using one-way ANOVA followed by Bonferroni’s post-hoc comparisons tests (ns: not significant, *** *p* ≤ 0.001 and **** *p* ≤ 0.0001).

Furthermore, as miRNA-574 was one of a few miRNAs that have been identified in the CHO genome, we were interested whether relative mature miRNA-574-3p expression in CHO-ETN and CHO-EPO stable clones are comparable with pri-miR-574 and pri-miR-143 Chinese hamster flanking sequences. To address this question, we amplified Chinese hamster endogenous pri-miR-574 (cgr-pri-miR-574) from the CHO genome and prepared CHO-ETN and CHO-EPO stable clones overexpressing miRNA-574-3p. RT-PCR analysis showed comparable levels of mature miRNAs expression from two Chinese hamster flanking sequences (Figure 4). Therefore, based on our findings, we suggest a new strategy in CHO cell engineering for any miRNA stable expression using the Chinese hamster pri-miR-143 flanking motifs.

### 3.4. Stable Expression of miR-574-3p Increases the Level of Recombinant mRNA, Protein Production and the Anti-Apoptotic Effect in CHO Cells 

**Figure 5:**
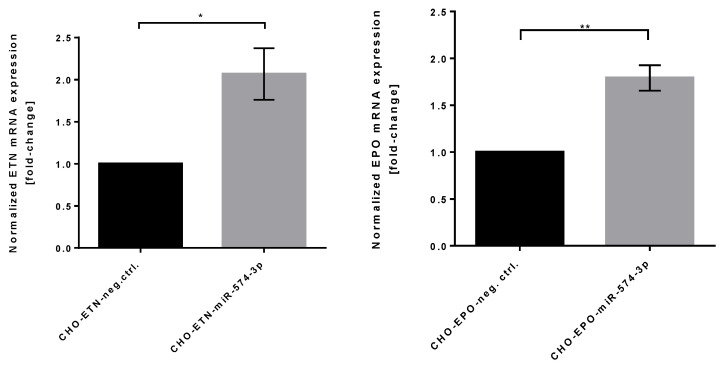
Relative ETN and EPO mRNA levels in stable overexpressing miR-574-3p CHO clones. Quantitative RT-PCR analysis of the relative levels of ETN and EPO mRNAs. MMADHC mRNA was used for normalization and results are expressed as the mean of fold change in expression in CHO-ETN/EPO-miR-574-3p cells compared to the control cells (SEM as error bars, *n* = 3). Statistical analysis is performed using *t*-test followed by Bonferroni post hoc test (* *p* ≤ 0.05, ** *p* ≤ 0.01).

Further, we investigated ETN and EPO gene expression levels in two miRNA-574-3p overexpressing stable CHO clones by quantitative real-time RT-PCR in order to address whether the higher productivity was mainly due to the mRNA transcript abundance. In order to determinate relative changes in EPO and ETN mRNA transcript abundance, we used MMADHC for normalization as it has been identified as an optimal control reference gene for qPCR analysis of CHO cell mRNAs [43]. As shown in Figure 5, overexpression of miR-574-3p induced an average 2-fold increase in mRNA of EPO and ETN in CHO cells indicating that miRNA-574-3p regulation of ETN/EPO synthesis involves transcription rather than translation/secretion. 

**Figure 6:**
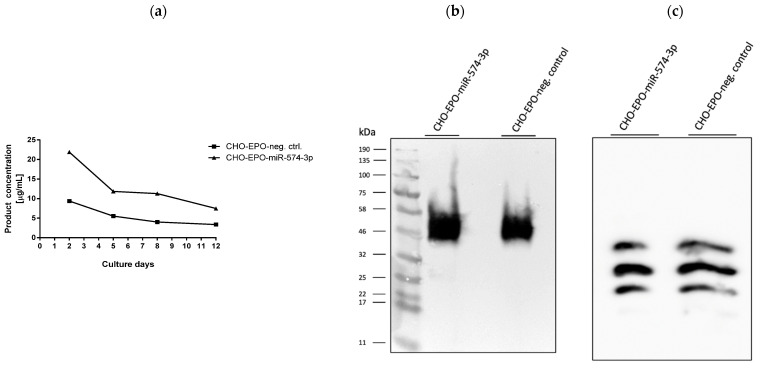
Effect of stable overexpression of miR-574-3p on EPO production. (**a**) EPO production in serum-free media over 12-days. (**b**) Western blot analysis of EPO produced in serum-free media in stable overexpressing miR-574-3p and CHO control cells (anti-EPO antibody). (**c**) Isoelectric focusing pattern of differently glycosylated EPO isoforms purified from CHO-EPO-miR-574-3p cells and CHO-EPO control cells.

In order to test the effect of miR-574-3p in the scaled-up setting, we decided to select a stable CHO-EPO single clone and grow it in serum-free production media for 12 days. During the cultivation time, the media was replaced every three days and the EPO concentration was determined after affinity chromatography purification (Figure 6a). At the end of production, total EPO was further purified through anion exchange chromatography. Stable miR-574-3p overexpressing cells yielded more than 2-fold of EPO compared to the established and well-characterized control cells. As EPO biological activity is heavily dependent on glycosylation [44], we decided to look at the isoform profile of EPO purified from miR-574-3p versus control cells by IEF. The quality of purified EPO was comparable in both the miR-574-3p overexpressing cells and the control cells as estimated by the SDS-PAGE (Figure 6b) and IEF analysis (Figure 6c). Our results show that miR-574-3p is a good candidate to boost heterologous protein production in CHO cells without impact on its quality.

**Figure 7:**
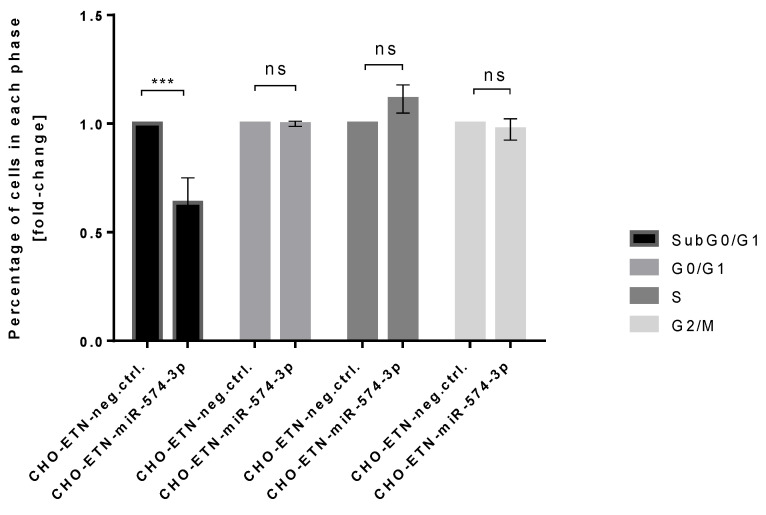
miR-574-3p overexpression induces less apoptosis in CHO-ETN cells. Cell-cycle analysis by flow cytometry after BrdU and propidium iodide staining of CHO-ETN cells stably expressing miR-574-3p, shows less subG0/G1 cells in comparison to the control cells after 4 days of cultivation. All data is expressed as the mean ± SEM. (ns: not significant, *** *p* ≤ 0.001, *t*-test).

In addition, we tested whether miR-574-3p interferes with the cell cycle in the CHO production cell line, as it is known that the longer lasting period of cells in the G1 phase positively correlates with protein production. Therefore, we decided to quantify cell cycle phases of miRNA-574-3p versus control cells, by propidium iodide staining and analysis by flow cytometry. Cell cycle analysis showed no difference in the percentage of cells in any phase of cell cycle in miR-574-3p CHO-ETN versus control cells. The only significant difference was observed in the apoptotic and early apoptotic fractions (subG0/G1) where miR-574-3p shows an anti-apoptotic effect (Figure 7). 

### 3.5. Validation of miR-574-3p Targets in CHO Cells 

**Figure 8:**
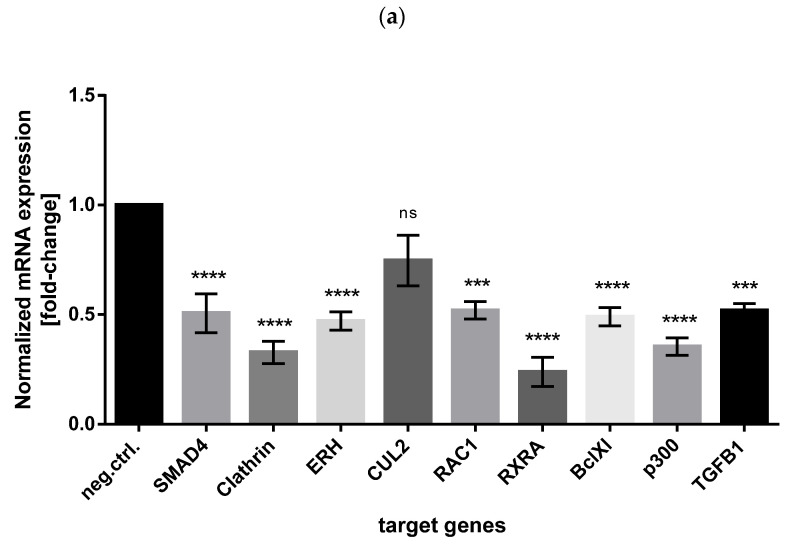

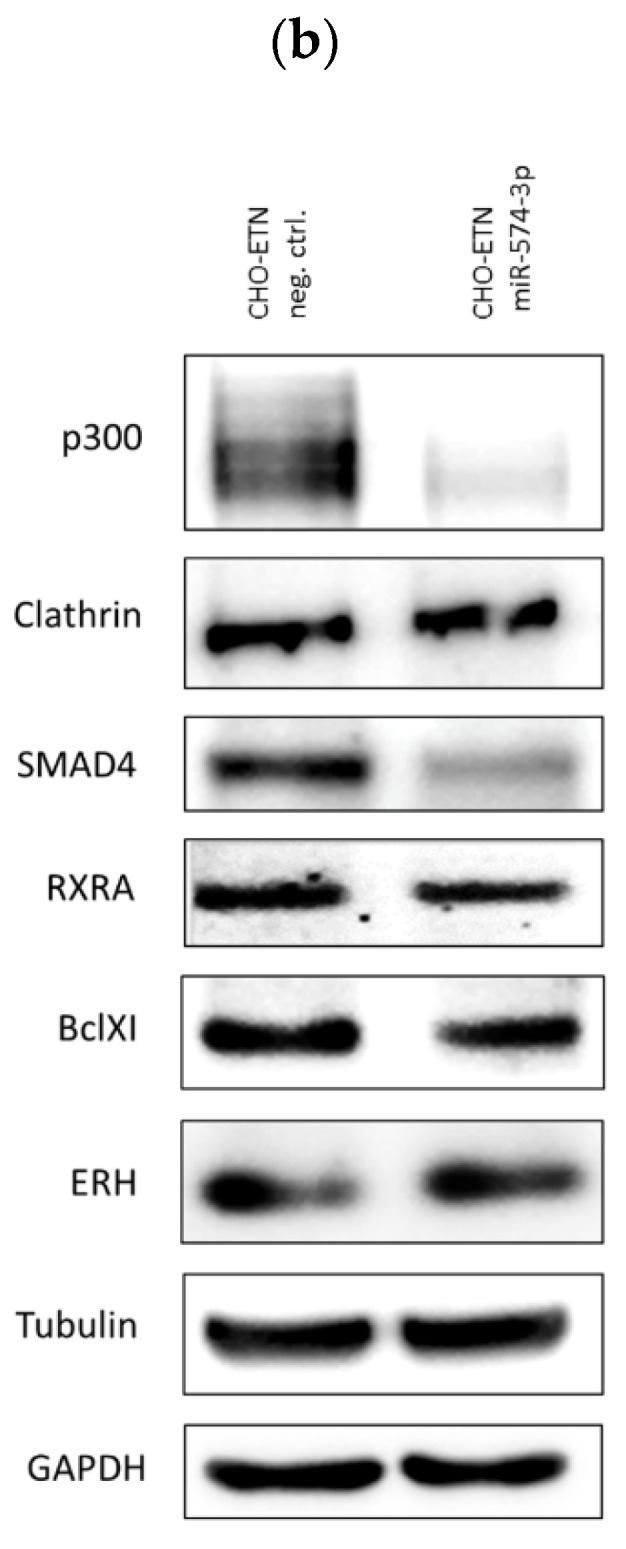
(**a**) Relative mRNA levels of nine annotated miR-574-3p target genes in CHO-ETN cells overexpressing miR-574-3p shown as fold-change relative to the control cells and normalized to MMADHC mRNA. Data is presented as the mean of three independent experiments ± SEM. Statistical analysis was done using one-way ANOVA followed by Bonferroni’s post-hoc comparisons tests (ns: not significant, *** *p* ≤ 0.001 and **** *p* ≤ 0.0001) (**b**) Western blot analysis shows downregulation of p300, SMAD4 and RXRA in CHO-EN stable cells overexpressing miR-574-3p.

Searching the miRTarBase database for experimentally validated miR-574-3p-targets, we checked whether CUL2, RAC1, p300, RXRA, SMAD4, TGFB1, Clathrin, ERH and BclXl genes were downregulated in miR-574-3p overexpressing CHO cells. Relative mRNA levels of eight out of nine selected annotated miR-574-3p target genes showed statistically significant downregulation in CHO-ETN cells overexpressing miR-574-3p (Figure 8a). Western blot analysis confirmed significant downregulation of at least five selected target proteins: p300 (14.7% of the control cells), Clathrin (82.4% of the control cells), SMAD4 (44.7% of the control cells), BclXl (79.9% of the control cells), ERH (100% of the control cells) and RXRA (67.4% of the control cells) (Figure 8b). We therefore identified p300, SMAD4 and RXRA as the most prominent downregulated proteins upon miR-574-3p overexpression in CHO cells.

### 3.6. Effect of p300 and SMAD4 Knockdown on Heterologous Gene Expression

**Figure 9:**
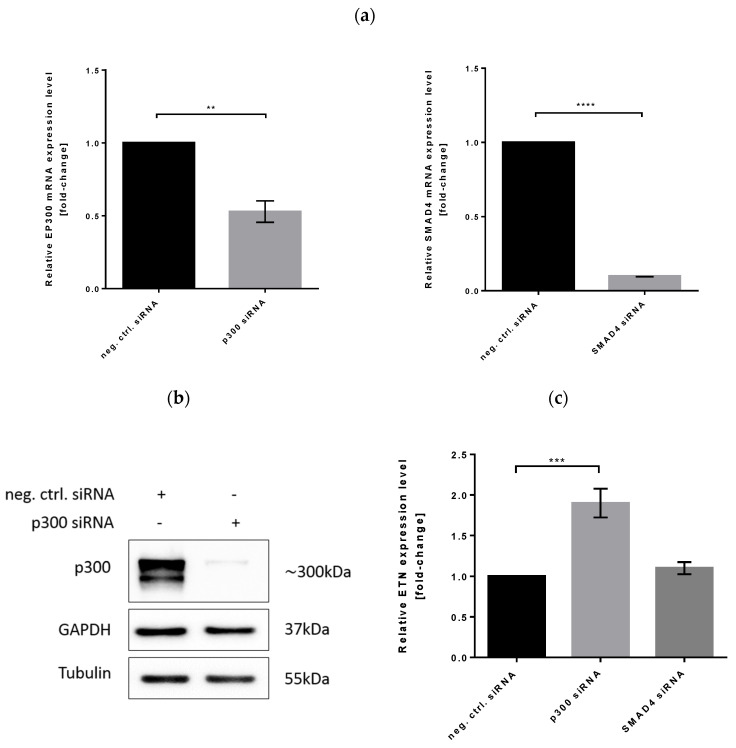
Impact of p300 and SMAD4 knockdown on ETN transcription in CHO-ETN cells. (**a**) Quantitative real-time RT-PCR analysis of relative levels of p300 and SMAD4 mRNAs in siRNA transfected CHO-ETN cells. (**b**) Western blot analysis shows significant downregulation of p300 in CHO-ETN cells. The graph shows the mean ± SD from three independent experiments (unpaired *t*-test; ** *p* ≤ 0.01, *** *p* ≤ 0.001 and **** *p* ≤ 0.0001) (**c**) Quantitative real-time RT-PCR analysis of relative levels of ETN and SMAD4 in siRNA transfected CHO-ETN clones. MMADHC mRNA was used for normalisation and results are expressed as the mean of fold change in expression in CHO-ETN cells compared to the control cells (SEM as error bars, *n* = 3).

To examine the functional role of p300 and SMAD4 proteins in increased ETN transcription, we performed a loss-of-function study in CHO cells using p300 and SMAD4 siRNAs. The mRNA and protein level of p300 and SMAD4 was significantly lower in siRNA transfectants compared to controls (Figure 9a,b). Once we established an efficient interference, we looked at the relative expression level of ETN by quantitative real-time RT-PCR analysis. We found that ETN expression was significantly increased in CHO cells transfected with p300 siRNAs in comparison to the control cells but not in SMAD4 silenced cells (Figure 9c). An average 2-fold increase was comparable to the one seen in miR-574-3p overexpressing CHO-ETN cells, thus providing evidence for p300 as a miR-574-3p target responsible for increased protein production in CHO cells.

### 3.7. miR-574-3p Overexpression Induces p53 Downregulation in CHO Cells

**Figure 10:**
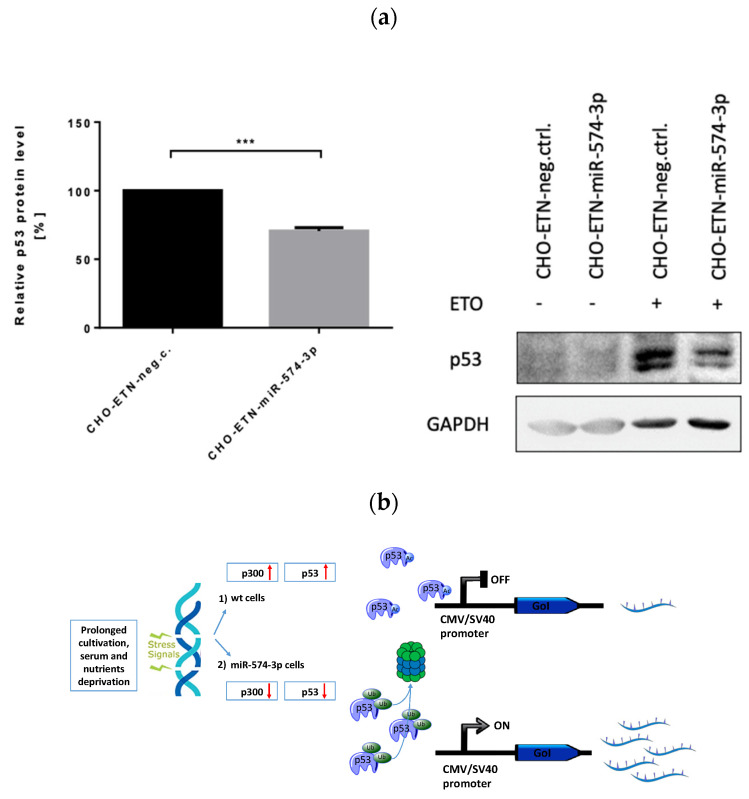
MiR-574-3p downregulates p53. (**a**) CHO cells stably expressing miR-574-3p were exposed to etoposide (ETO 250 μM) for 16 h. Western blot analysis shows significant downregulation of p53 in CHO-ETN cells. The graph shows the mean ± SEM from three independent experiments (unpaired *t*-test, *** *p* < 0.001) (**b**) Proposed miR-574-3p mode of action through p300 and p53 downregulation 1. In the wt cells, under different stresses, such as prolonged cell cultivation and nutrient deprivation, p300 is activated as well as p53. p300 is a major acetyltransferase for p53 that acetylates p53 and therefore stabilizes it. p53 suppresses viral promoter activity interfering with the basal transcription machinery. Upon miR-574-3p overexpression, p300 is downregulated resulting in a lower p53 acetylation, destabilization and degradation removing viral promoter repression and increasing heterologous gene transcription.

p300 and SMAD4 have been associated with transcriptional regulation where p300 acetyltransferase acetylates around 100 protein substrates and binds over 400 protein ligands [45]. SMAD4 is one of the protein binding partners for p300 [46] as well as RXRA [47]. p300 is known to acetylate RXRA on lysine 145 [47] and acts as a major acetyltransferase for transcription factor p53 that acetylates p53 in a C-terminal domain, known to be critical for the regulation of p53 DNA binding activity [48,49]. It has been demonstrated that p53 acetylation on its C-terminus by p300 enhances its DNA-binding affinity for promoters. These data suggest that C-terminal acetylation alters p53 conformation leading to increased DNA-binding activity. Overall, p53 acetylation strongly correlates with protein stabilization while ubiquitination, which occurs on the same lysine residues, causes the degradation of p53 [50]. There is considerable evidence that demonstrates p53 represses transcription from several cellular and viral promoters including CMV and SV40 through interference with TATA motif and basal transcription machinery [51,52,53,54,55,56,57,58]. Therefore, we aimed to investigate whether downregulation of p300 by miR-574-3p decreases p53 level alleviating CMV promoter repression and consequently increasing recombinant protein expression. Thus, we checked the p53 expression level in miR-574-3p overexpressing CHO-ETN cells in comparison to the control cells (ETN gene is under the CMV promoter). In order to stabilize and visualize p53 in the Western blot, we treated the cells with 250 μM etoposide. Western blot analysis demonstrated that the p53 protein level in CHO-ETN cells overexpressing miR-574-3p decreased by about 30% in comparison to CHO-ETN control cells (Figure 10). 

On the basis of the results obtained, we propose a model of miR-574-3p action that involves p300 and p53 as effector proteins. Overexpression of miR-574-3p induces a drastic reduction in p300 expression that consequently results in p53 downregulation, destabilization and degradation. Lower p53 levels de-repress the CMV promoter and result in higher heterologous protein expression (Figure 10b).

## 4. Discussion

The construction of highly productive CHO cell lines is still a challenging task in biotech engineering. Cell engineering can substantially benefit from miRNAs as a tool which regulates nearly all biotechnologically important cellular processes such as proliferation, metabolism, cell death, transcription and protein production [59]. Several research studies provide evidence that miRNAs are critically important in recombinant protein production in CHO cells. Bort et al. studied the miRNA and mRNA profile of the CHO-K1 cell line where over 1400 mRNAs and 100 miRNAs were identified as differentially expressed in various growth phases [60]. Several research groups compared the miRNA expression profile of low and high producing CHO cell lines [23,61,62], in response to temperature shifts from 37 °C to 30 °C [26], various media compositions and different culture systems [63] in order to identify potential engimiRs or biomarkers and cell engineering targets. Maccani et al. highlighted that 83 miRNAs were differentially expressed and that overall miRNA expression levels were increased in high-producers [62]. Furthermore, Klanert et al. reported 12 miRNAs that positively regulate growth of CHO-K1, CHO-S and CHO-DUKXB11 cell lines [63]. Our study aimed to identify miRNAs that are able to boost production in CHO cells in a product-independent manner. Despite the fact that a miRNAs library from mouse cells is evolutionarily closer to Chinese hamsters, we decided to use a human miRNAs library to open up the possibility of identifying some miRNAs with conserved targets and functions not yet found in Chinese hamsters. Indeed, we were able to detect a positive effect on recombinant protein production for as yet unknown miRNAs in the CHO genome, such as miR-18b-3p, miR-521-1-3p, miR-3667-5p and miR-3939-3p. Moreover, we identified miR-574-3p as the best candidate amongst more than 2000, which is able to increase cell-specific and overall production of EPO and ETN in CHO cells. While initial screen with 2042 miRNA mimics revealed 1015 miRNAs with enhanced cell-specific EPO productivity and 370 miRNAs with higher volumetric EPO productivity we focused our attention on the best 124 performers for a secondary screening in CHO-EPO and CHO-ETN cells. In fact, the majority of the 124 miRNAs that we tested could be validated to enhance productivity for both CHO clones. In selecting only 124 we are aware that we may be excluding some other impactful miRNA candidates. Therefore, further potential lies in validation experiments with a broader range of identified miRNAs for both recombinant proteins production.

miRNA target recognition is determined by base pairing between the eight-base seed sequence of the miRNA from the 5’ end and the mRNA transcript [6]. Kehl et al. found a positive correlation between seed similarity in miRNAs and target gene/pathway similarity [64]. Interestingly multiple sequence alignment of seven miRNAs selected in our screening (miR-143-3p, miR-18b-3p, miR-132-5p, miR-521-1-3p, miR-574-3p, miR-3667-5p and miR-3939-3p) shows a consensus seed. ACGC.CA that might indicate its involvement in gene silencing activity of similar targets (Supplementary Figure S3). 

Whilst transient overexpression is useful for identification of promising miRNAs for cell engineering, generation of stable overexpressing cells is necessary in order to address miRNA biogenesis complexity. As four out of seven miRNAs of interest were not present in the CHO miRBase, we decided to use human pri-miRNA homologs and their flanking sequences for stable clone generation. Mature miR-18b-3p, miR-3667-5p and miR-3939-3p from constructs having human flanking regions were not detected in the cytoplasm of CHO cells contrary to endogenous ones. Our results suggest that human flanking sequences, which are not completely homologous to those in hamsters are not efficiently processed by Drosha. It has been previously reported that one of seven miRNAs of interest, miR-143-3p, can induce a hyper-productive phenotype in CHO cells [29]. Therefore, we used a CHO pri-miRNA-143 scaffold to drive the expression of the other miRNAs of interest and to demonstrate that all six pri-miRNA constructs were successfully processed by Drosha and exported from the nucleus. Although the work of Zeng and Cullen showed that efficient Drosha processing of a pri-miRNA in vitro, on the long flanking ssRNA sequences to stem-loop structure, occurs in a sequence-independent manner [40], the principal findings of our study is that pri-miRNA processing in hamster flanking sequences is more efficient than that of human sequences in CHO cells. Similar observations were provided by Klanert et al. who demonstrated inefficient processing of chimeric pri-miRNAs |(mature CHO miRNA with mouse pri-miR-155 flanking and loop sequences) in comparison with endogenous miR-221 and miR-222 constructs [17]. In addition to the importance of the flanking sequence in pri-miRNA metabolism, and in correlation with the findings of Roden et al. [38], our data suggests that several structural features, such as preserved hairpin stem length, apical loop and the number and location of bulges along the stem, contribute to efficient pri-miRNA processing. Additionally, we propose that our design strategy of site-directed mutagenesis maintaining hamster-specific flanking sequences of pri-miR-143, the length of the miR-143 stem, the location and size of bulges within the stem, allows for the replacement of a mature miRNA-143-3p sequence with any miRNA of choice in CHO cells. 

The stable overexpression of miRNA-574-3p demonstrated an average increase of 30–40% in recombinant protein production after 4 days of cultivation in line with several other studies with miR-17, miR-19b or miR-92a [23]. We also demonstrated that this is mainly due to mRNA transcript abundance. A positive trend in cell survival and protein production was even more pronounced in a larger scale production batch after prolonged cultivation (12 days) in serum-free media (which doubled the EPO expression compared to the control cells). 

To date, no information is available about miR-574-3p function in CHO cells. Identifying complex and diverse miRNA-mRNA target interactions is still a major challenge that is further hampered by the unavailability of data for the Chinese hamster genome. Hence, while searching for experimentally validated miR-574-3p-targets in the literature, we assumed a high preservation of miRNA and target mRNA interactions between Chinese hamster, mouse and human cells. We demonstrated a significant downregulation of several proteins such as p300, SMAD4 and RXRA following miRNA-574-3p overexpression in CHO cells. All three transcripts have miR-574-3p putative binding sites and SMAD4 and RXRA are known as p300 binding partners [46,47]. Our loss-of-function experiment, where endogenous p300 expression was silenced by siRNA, demonstrated enhanced ETN production. This suggests that miRNA-574-3p might regulate p300 acetyltransferase directly involved in recombinant protein expression. Interestingly, the work of Zebing Hu et al., demonstrated that p300 was a direct target of miR-132-3p as well, which was one of the seven miRNAs identified in our screening [65]. While it is widely reported that p300 is associated with cell-cycle pathway and has pro-apoptotic function in cells under different types of stress [66], we asked the question whether miR-574-3p induced p300 downregulation might have an effect on cell cycle and apoptosis in CHO cells overexpressing ETN. Our cell cycle experiment, with flow cytometry after BrdU and propidium staining, indeed shows that miR-574-3p has an anti-apoptotic effect in CHO-ETN cells (Figure 7). 

Viral promoters such as CMV and SV40 frequently drive recombinant gene expression in biopharmaceutical processes. Indeed, our two constructs, ETN and EPO, include CMV and SV40 promoters, respectively. p53 is able to repress both promoters interfering with basal transcription machinery and the stable binding of TBP and TFIIA to the TATA motif, required for efficient transcriptional initiation [52,53,57,67]. Moreover, p53 is known as a p300-binding partner that interacts with acetyltransferase p300 to become acetylated [45]. In response to cellular stresses, such as DNA damage, p53 activity is modulated by acetylation, which is responsible for its stabilization [50]. Furthermore, it has been shown that acetylation of p53 abrogates its ubiquitination by Mdm2 and therefore its ubiquitination-dependent proteasome proteolysis [68]. In investigating the p53 level in miRNA-574-3p overexpressing cells versus control, we suggest that p300 downregulation decreases p53 acetylation. This results in p53 destabilization, degradation and releases repression of the viral promoter. 

There is increasing evidence that the control of transcription and regulation of mRNA stability are interconnected. One of the other ways in which p300 acetyltransferase interferes with protein production in miRNA-574-3p CHO cells might be through the regulation of mRNA stability. p300, and its closely related acetyltransferase CBP, promote the removal of poly (A) tails from mRNAs and mRNA degradation through acetylation of the CCR4-CAF1-NOT deadenylation complex [69]. This complex plays a central role in the decay of eukaryotic mRNAs [70]. In fact, pharmacological inhibitors of p300 and CBP were found to stabilize poly (A) mRNAs suggesting that protein acetylation directly affects the RNA degradation machinery. 

Identifying p300 as a main miR-574-3p target opens the door to the potential to influence a myriad of cellular functions that could be involved in enhanced CHO cell productivity. This is because p300 is essential to many cellular signaling pathways through its numerous protein−protein interactions which integrate diverse signals in the cell. Taken together, our findings suggest that miR-574-3p may be a valuable tool for the improvement of recombinant protein productivity in CHO cell lines but in order to prove its worth we would need to test it with a wider range of recombinant proteins of pharmaceutical interest such as monoclonal antibodies. Further testing in high producer CHO suspension cell lines would enable us to ascertain the applicability of miR-574-3p in industrial settings. The results from the present study help us to understand the biogenesis of miRNAs and the regulatory mechanism of miR-574-3p in CHO cells as a necessary prerequisite for the successful application of miRNAs in cell line engineering.

## Supplementary Materials

The following are available online at https://www.mdpi.com/article/10.3390/biom11081125/s1, Figure S1: The effect of 124 miRNA mimics transiently transfected in CHO-EPO and CHO-ETN cells. Normalized volumetric productivities are presented as fold change relative to the respective negative control, Figure S2: (a) PCR analyses of genomic DNA of CHO-ETN stable cells, engineered with human pri-miRNA-143, pri-miRNA-18b, pri-miRNA-521, pri-miRNA-574, pri-miRNA-3667 and pri-miRNA-3939 using forward CMV promoter and reverse primiRNAs specific primers, confirm the presence of the human pri-miRNAs (b) Sequence alignment of hsa-pri-miR-143 and cgr-pri-miR-143 sequences using EMBOSS WATER software. The mature miRNA sequence is highlighted in grey, Figure S3: Multiple sequence alignment of miR-143-3p, miR-18b-3p, miR-132-5p, miR-521-1-3p, miR-574-3p, miR-3667-5p and miR-3939-3p, Table S1: Primers used to amplify pri-miRNA sequences of Homo sapiens (hsa) and Cricetulus griseus (cgr), Table S2: Primers for site-directed mutagenesis, Table S3: Primers usied for qPCRs; Table S4: Primers used for detection of primary miRNA transcripts, Table S5: Oligonucleotides for siRNA generation (siRNAs).
