# Supplementary material for: High Throughput miRNA Screening Identifies miR-574-3p Hyperproductive Effect in CHO Cells
Source: Biomolecules. 2021 Jul 30;11(8):1125. doi: 10.3390/biom11081125 (PMC8392531; doi:10.3390/biom11081125)
Supplement: Supplementary file 1 [file biomolecules-11-01125-s001.zip › biomolecules-1275993-supplementary.pdf]

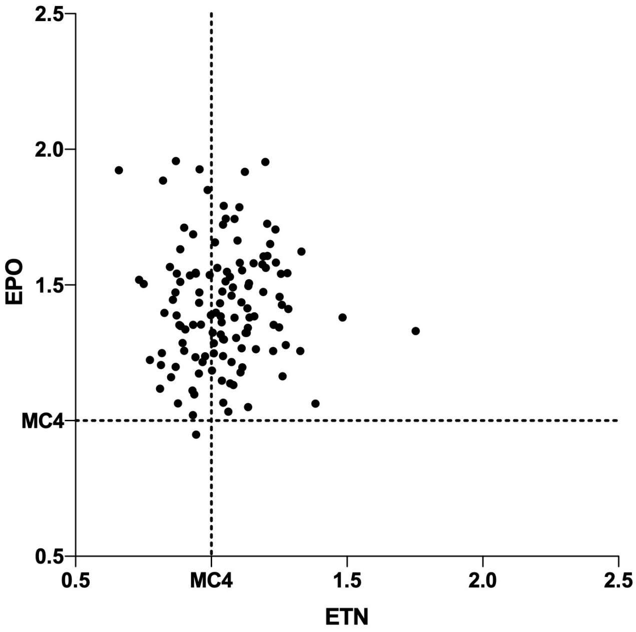

Supplementary Figure S1: The effect of 124 miRNA mimics transiently transfected in CHO-EPO and CHO-ETN cells. Normalized volumetric productivities are presented as fold change relative to the respective negative control.

a)

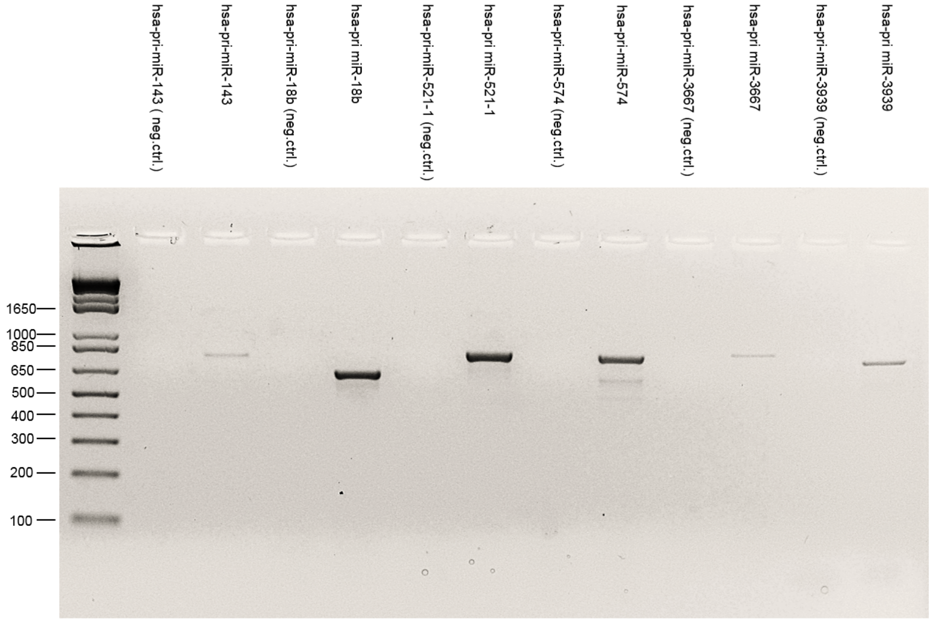

b)

```
hsa-pri-mir143     11 CCAGAGCTGGAGAGGT-GGAGCCCAGGTCCCCT-------CTAACACCCC     52
                      |.||.|..|.|.|||| |||| .|||| ||.||       ||.||||.||
cgr-pri-mir143      1 CAAGGGAAGAACAGGTGGGAG-GCAGG-CCACTACACATGCTCACACTCC     48

hsa-pri-mir143     53 TTCTCCTGGCCAGGTTGGAGTCCCGCCACAGGCCACCAGAGCG---GAGC     99
                      ||.|||||.|||    |||..||.|||.|||  |.||||.|||   |.||
cgr-pri-mir143     49 TTTTCCTGCCCA----GGAAGCCAGCCGCAG--CCCCAGTGCGCATGTGC     92

hsa-pri-mir143    100 AGCGCAGCGCCCTGTCTCCCAGCCTGAGGTGCAGTGCTGCATCTCTGGTC    149
             |         |||.|||| .|||||||||||||||||||||||||||||||||||||||
cgr-pri-mir143     93 -GCGGAGCG-TCTGTCTCCCAGCCTGAGGTGCAGTGCTGCATCTCTGGTC    140

hsa-pri-mir143    150 AGTTGGGAGTCTGAGATGAAGCACTGTAGCTCAGGAAGAGAGAAGTTGTT    199
                      ||||||||||||||||||||||||||||||||||||||.|||||||||||
cgr-pri-mir143    141 AGTTGGGAGTCTGAGATGAAGCACTGTAGCTCAGGAAGGGAGAAGTTGTT    190

hsa-pri-mir143    200 CTGCAGCCATCAGCCTGGAAGTGGTAAGTGCTGGGGGGTTGTGGGGGGCC    249
                      |||||||||||| ||.|||.||||||||||.|     |.|||||||
cgr-pri-mir143    191 CTGCAGCCATCA-CCAGGACGTGGTAAGTGTT-----GATGTGGGG----    230

hsa-pri-mir143    250 ATAACAGG-AAGGACAGAGTGTTTCCAGACTCCATACTATCAG-CCACTT    297
                       ||.|||| ..|||.||   |||   ||||||||..|.|.||| ||| |.
cgr-pri-mir143    231 -TAGCAGGCCCGGATAG---GTT---AGACTCCACTCCAGCAGTCCA-TG    272

hsa-pri-mir143    298 GTGAT    302
                      |||||
cgr-pri-mir143    273 GTGAT    277
```

Supplementary Figure S2: a) PCR analyses of genomic DNA of CHO-ETN stable cells, engineered with human pri-miRNA-143, pri-miRNA-18b, pri-miRNA-521, pri-miRNA-574, pri-miRNA-3667 and pri-miRNA-3939 using forward CMV promoter and reverse pri-miRNAs specific primers, confirm the presence of the human pri-miRNAs b) Sequence alignment of hsa-pri-miR-143 and cgr-pri-miR-143 sequences using EMBOSS WATER software. The mature miRNA sequence is highlighted in grey.

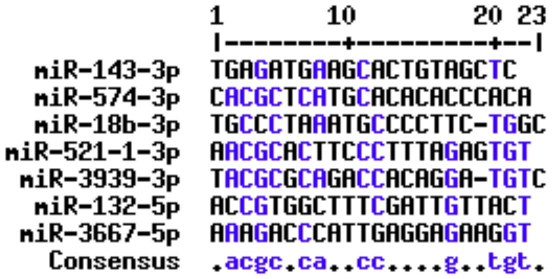

Supplementary Figure S3: Multiple sequence alignment of miR-143-3p, miR-18b-3p, miR-132-5p, miR-521-1-3p, miR-574-3p, miR-3667-5p and miR-3939-3p.

**Primer name**	**Sequence**
Hsa-pri-mir-143 fw	5’-TATGGATCCAAGGTTTGGTCCTGGGTGCTCAAAT-3’
Hsa-pri-mir-143 rv	5’-AAAGAATTCTGCTAACGCCTCATGCTAAGATGG-3’
Hsa-pri-mir-18b fw	5’-TATGGATCCCCATGGTGATTTAGTCAATGGCTAC-3’
Hsa-pri-mir-18b rv	5’-AAAAAGAATTCAGCACTTTGGTACTACTAGGACCCA-3’
Hsa-pri-mir-521-1 fw	5’-TATGGATCCTCAGGAGGGTTGCCCCTGCATGAA-3’
Hsa-pri-mir-521-1 rv	5’-AAAAAGAATTCAGGCAGAAGAATGGCGTGAACCTGG-3’
Hsa-pri-mir-574 fw	5’-TATGGATCCTACTCGGCCGCCTGAGCGGTAAGA-3’
Hsa-pri-mir-574 rv	5’-AAAAAGAATTCTGGGACGAGGCCTCTGTCTTACAG-3’
Hsa-pri-mir-3667 fw	5’-TATGGATCCCTTTTGAGATGCTGACTTTCTGTG-3’
Hsa-pri-mir-3667 rv	5’-AAAAAGAATTCAGGATGCTTCTACCAATGAGGA-3’
Hsa-pri-mir-3939 fw	5’-TATGGATCCCAGGCCTCAGCTTTCAGCTTAA-3’
Hsa-pri-mir-3939 rv	5’-AAAAAGAATTCATATGTGTGTACATACCCACAGACA-3’
BLOCKnc-to-miRVec-BamHI fw	5’-TTTTTGGATCCTGGAGGCTTGCT-‘3

BLOCKnc-to-miRVec-EcoRI rv	5’-TATATAGAATTCTAGATCAACCACTTTGTACAA-‘3
Cgr-pri-mir-574 fw	5’-TATGGATCCACTCGGCGGCCAAGCGGTAAGAG-3’
Cgr-pri-mir-574 rv	5’-AAAAAGAATTCAGGGCTAGGGCAGGCACACTCTAGG-3’

Supplementary Table S1. Primers used to amplify pri-miRNA sequences of *Homo sapiens* (hsa) and *Cricetulus griseus* (cgr)

**Primer name**	**Sequence**
QC-miR-18b-3p fw	5’-GTGCGCGGAGCGTCTGTCTCCCAGCCGCCACAAGGGGCATATAGGGCTGGTCAGTTGGGAGTCTGCCCTAAATGCCCCTTCTGGCGGAAGGGAGAAGTTGTTCTGCAGCCA-‘3
QC-miR-18b-3p rv	5’-TGGCTGCAGAACAACTTCTCCCTTCCGCCAGAAGGGGCATTTAGGGCAGACTCCCAACTGACCAGCCCTATATGCCCCTTGTGGCGGCTGGGAGACAGACGCTCCGCGCAC-‘3
QC-miR-132-5p fw	5’-GTGCGCGGAGCGTCTGTCTCCCAGCCAGTATCAATCGAAACCCACGGAGGTCAGTTGGGAGTCACCGTGGCTTTCGATTGTTACTGGAAGGGAGAAGTTGTTCTGCAGCCA-‘3
QC-miR-132-5p rv	5’-TGGCTGCAGAACAACTTCTCCCTTCCAGTAACAATCGAAAGCCACGGTGACTCCCAACTGACCTCCGTGGGTTTCGATTGATACTGGCTGGGAGACAGACGCTCCGCGCAC-‘3
QC-miR-521-1-3p fw	5’-GTGCGCGGAGCGTCTGTCTCCCAGCCACACACTAAAGGGATGTGCGTAGGTCAGTTGGGAGTCAACGCACTTCCCTTTAGAGTGTGGAAGGGAGAAGTTGTTCTGCAGCCA-‘3
QC-miR-521-1-3p rv	5’-TGGCTGCAGAACAACTTCTCCCTTCCACACTCTAAAGGGAAGTGCGTTGACTCCCAACTGACCTACGCACATCCCTTTAGTGTGTGGCTGGGAGACAGACGCTCCGCGCAC-‘3
QC-miR-574-3p fw	5’-GTGCGCGGAGCGTCTGTCTCCCAGCCTGTGCGTGTGTGCAAGAGCGTCGGTCAGTTGGGAGTCCACGCTCATGCACACACCCACAGGAAGGGAGAAGTTGTTCTGCAGCCA-‘3
QC-miR-	5’-TGGCTGCAGAACAACTTCTCCCTTCCTGTGGGTGTGTGCATGAGC

574-3p rv	GTGGACTCCCAACTGACCGACGCTCTTGCACACACGCACAGGCTGGGAGACAGACGCTCCGCGCAC-‘3
QC-miR-3667-5p fw	5’-GTGCGCGGAGCGTCTGTCTCCCAGCCAAAGACCCATTGAGGAGAAGGTGGTCAGTTGGGAGTCACCTTCCTCTCCATGGGTCTTTGGAAGGGAGAAGTTGTTCTGCAGCCA-‘3
QC-miR-3667-5p rv	5’-TGGCTGCAGAACAACTTCTCCCTTCCAAAGACCCATGGAGAGGAAGGTGACTCCCAACTGACCACCTTCTCCTCAATGGGTCTTTGGCTGGGAGACAGACGCTCCGCGCAC-‘3
QC-miR-3939-3p fw	5’-GTGCGCGGAGCGTCTGTCTCCCAGCCGACAACCTGTGGTCAGCGCGTTGGTCAGTTGGGAGTCTACGCGCAGACCACAGGATGTCGGAAGGGAGAAGTTGTTCTGCAGCCA-‘3
QC-miR-3939-3p rv	5’-TGGCTGCAGAACAACTTCTCCCTTCCGACATCCTGTGGTCTGCGCGTAGACTCCCAACTGACCAACGCGCTGACCACAGGTTGTCGGCTGGGAGACAGACGCTCCGCGCAC-‘3

Supplementary Table S2. Primers for site-directed mutagenesis

**Primer name**	**Sequence**
CLTC(Clathrin)-qPCR fw	5’-CGCTTGGCATCTACCCTTGTTC-3’
CLTC(Clathrin)-qPCR rv	5’-AGCAGACCTCCTTCCATGTTCG-3’
CUL2-qPCR fw	5’-TCAGCAGCGTATGGTAGCAGAC-3’
CUL2-qPCR rv	5’-TGGACACAGCACGGAGCAAG-3’
RAC1-qPCR fw	5’-TGTCCCAACACTCCCATCATCC-3’
RAC1-qPCR rv	5’-CGCTGAGCACTCCAGGTATTTG-3’
RXRA-qPCR fw	5’-CTACGGGCAGGCTGGAATGAG-3’
RXRA-qPCR rv	5’-GCTATGGAACGGTGGGAGAAGG-3’
Bcl2l1(BclXl)-qPCR fw	5’-TGACTGTGGCTGGTGTGGTTC-3’
Bcl2l1(BclXl)-qPCR rv	5’-GGAGGCGAGATGTGAGTAGGTG-3’
p300-qPCR fw	5’-CAGACACCAACACCACCAACAC-3’
p300-qPCR rv	5’-GCAGGAGCAGCAGGAATTGAAG-3’
SMAD4-qPCR fw	5’-CTGGACGAGCACCTGGAGAC-3’
SMAD4-qPCR rv	5’-ACACTGCCGCAAATCAAAGACC-3’
TGFB1-qPCR fw	5’-ACGGAGAAGAACTGCTGTGTGC-3’
TGFB1-qPCR rv	5’-GTTGGTTGTAGAGGGCGAGGAC-3’
ERH-qPCR fw	5’-GCAGGACTTACGCTGACTATG-3’
ERH-qPCR rv	5’-GCTGTTGGGATTCATTCTCTTC-3’
MMADHC-qPCR fw	5’-TGTCACCTCAATGGGACTGC-3’
MMADHC-qPCR rv	5’-CAGGTGCATCACTACTCTGAAAC-3’
GAPDH-qPCR fw	5’-GAAAGCTGTGGCGTGATGG-3’
GAPDH-qPCR rv	5’-TACTTGGCAGGTTTCTCCAG-3’

EPO-qPCR fw	5’-TGTGGATAAAGCCGTCAGTG-3’
EPO-qPCR rv	5’-GTGTCAGCAGTGATTGTTCG-3’
ETN-qPCR fw	5’-CGTGGAGTGGGAATCTAATGG-3’
ETN-qPCR rv	5’-GTGACAGTGACAGGCTCTTC-3’

Supplementary Table S3. Primers usied for qPCRs

Primer name	Sequence
pre-miRNA-18b fw	5’-TGCCAGAAGGGGCATTTAGG-3’
pre-miRNA-18b rv	5’-TGTGTTAAGGTGCATCTAGTGC-3’
pre-miRNA 3667 fw	5’-AAAGACCCATGGAGAGGAAG-3’
pre-miRNA 3667 rv	5’-CTTATCTAGCTTCTCTGAGGAT -3’
pre-miRNA 3939 fw	5’-AAGCCAGTGTGGACATCCTG-3’
pre-miRNA 3939 rv	5’-GCTTCCAAAGGCCTCTGTG-3’

Supplementary Table S4. Primers used for detection of primary miRNA transcripts

Primer name	Sequence
p300 siRNA	5’- [UUGGACUACCCUAUCAAGUAA]TT-3’
	5’- [UUACUUGAUAGGGUAGUCCAA]TT-3’
SMAD4 siRNA	5’- [GGUGGAGAAAGUGAAACGU]TT-3’
	5’- [ACGUUUCACUUUCUCCACC]TT-3’

Supplementary Table S5. Oligonucleotides for siRNA generation (siRNAs)
